# Assessing the Biocontrol Potential of *Clonostachys* Species Isolated as Endophytes from *Coffea* Species and as Mycoparasites of *Hemileia* Rusts of Coffee in Africa

**DOI:** 10.3390/jof9020248

**Published:** 2023-02-13

**Authors:** Miraine Kapeua-Ndacnou, Lucas Magalhães de Abreu, Davi Mesquita de Macedo, Thaisa Ferreira da Nóbrega, Caio Mattos Pereira, Harry Charles Evans, Robert Weingart Barreto

**Affiliations:** 1 Departamento de Fitopatologia, Universidade Federal de Viçosa, Viçosa 36570-900, Brazil; 2 Regional Biocontrol and Applied Microbiology Laboratory, Institute of Agricultural Research for Development, Yaoundé P.O. Box 2067, Cameroon; 3 CAB International, Bakeham Lane, Egham TW20 9TY, UK

**Keywords:** biological control, Bionectriaceae, coffee leaf rust, phylogeny, plant disease, taxonomy

## Abstract

During surveys conducted in South America and Africa to identify natural fungal enemies of coffee leaf rust (CLR), *Hemileia vastatrix*, over 1500 strains were isolated, either as endophytes from healthy tissues of Coffea species or as mycoparasites growing on rust pustules. Based on morphological data, eight isolates—three isolated from wild or semiwild coffee and five from *Hemileia* species on coffee, all from Africa—were provisionally assigned to the genus *Clonostachys*. A polyphasic study of their morphological, cultural and molecular characteristics—including the Tef1 (translation elongation factor 1 alpha), RPB1 (largest subunit of RNA polymerase II), TUB (β-tubulin) and ACL1 (ATP citrate lyase) regions—confirmed these isolates as belonging to three species of the genus *Clonostachys*: namely *C. byssicola*, *C. rhizophaga* and *C. rosea f. rosea*. Preliminary assays were also conducted to test the potential of the *Clonostachys* isolates to reduce CLR severity on coffee under greenhouse conditions. Foliar and soil applications indicated that seven of the isolates had a significant effect (*p* < 0.05) in reducing CLR severity. In parallel, in vitro tests that involved conidia suspensions of each of the isolates together with urediniospores of *H. vastatrix* resulted in high levels of inhibition of urediniospore germination. All eight isolates showed their ability to establish as endophytes in *C. arabica* during this study, and some proved to be mycoparasites of *H. vastatrix*. In addition to reporting the first records of *Clonostachys* associated with healthy coffee tissues and with *Hemileia* rusts of coffee, this work provides the first evidence that *Clonostachys* isolates have potential as biological control agents against CLR.

## 1. Introduction

Coffee is ranked as the most economically valuable agricultural commodity [1]. Only two species of the genus Coffea are of commercial relevance: *C. arabica* and *C. canephora* (Rubiaceae). Although Africa—the center of origin of the genus *Coffea*—plays a relatively small role in world coffee production, this crop is of great social relevance in that continent, as well as worldwide, for the income and employment it generates. It is produced mostly by smallholder communities [2]. However, there are increasing threats to coffee production from pests, diseases and adverse climatic conditions [3,4].

Amongst these limitations of coffee production is its most devastating disease: coffee leaf rust (CLR) caused by *Hemileia vastatrix* (Pucciniales: Zaghouaniaceae), a biotrophic fungus [5]. The recent report of CLR in Hawaii means that CLR has now spread to every significant coffee-growing region in the world [6]. The search for a sustainable, nonchemical and effective form of management for CLR is a major challenge [7,8]. Outbreaks of CLR in northern South America and Central America, starting in the early 2010s [3], have caused major economic and social distress in these regions and appear to be connected to increasing temperature (climate change), leading to the failure of the strategy of escaping *H. vastatrix* via planting coffee in highland situations. Biological control of *H. vastatrix* has, thus far, received relatively little attention.

Although there are several studies on potential antagonists of CLR, such as endophytic fungi that grow inside healthy coffee tissues [9,10,11,12,13] or mycoparasites that overgrow pustules of *H. vastatrix* [14,15,16,17,18], these have been focused predominantly in the Neotropics, where coffee and this rust are exotic species, and have yet to translate into practical advances in CLR management. Until recently, the mycobiota associated with *Coffea* in Africa as a source of antagonists of CLR, as well as of other pathogens that attack the crop, have been poorly studied. A notable exception is that of Mulaw [19]: a study that dealt with Trichoderma spp. isolated as endophytes from roots of *C. arabica* in Ethiopia and focused on their antagonism to *Fusarium xylarioides*, the causal agent of tracheomycosis.

In 2015, surveys were initiated in Africa for fungal antagonists of *H. vastatrix*, and an unexpectedly high diversity of fungi was revealed [20,21,22,23,24,25,26,27]. Amongst the more than 1500 isolates, a small number were assigned provisionally to the genus *Clonostachys* (Ascomycota: Hypocreales: Bionectriaceae). Here, we report on taxonomic studies to elucidate their identity, as well as on their potential as antagonists of *H. vastatrix*.

## 2. Materials and Methods

### 2.1. Survey and Isolation of Purported Antagonists of Hemileia vastatrix

Surveys for fungal antagonists of *H. vastatrix* were concentrated in Africa, focusing on Cameroon and Ethiopia—representing regions within the centers of origin of *C. canephora* and *C. arabica*, respectively—and Coffea species growing in wild or semiwild situations were targeted. Other *Hemileia* species, in addition to *H. vastatrix*, were also found on *Coffea* in these ecosystems. Details of the strategy and the isolation methodology employed to isolate endophytic fungi and mycoparasites are given in detail in previous publications [20,22]. Pure cultures of mycoparasitic fungi were obtained through direct transfer of spores, with a sterile needle, from colonized rust pustules onto PDA plates under a dissecting microscope. Endophyte isolations were performed in situ from thoroughly disinfested panels on either trunks or thick stems, with transfer of freshly exposed inner fragments onto 20% PDA plates supplemented with 10 mg/L of penicillin–streptomycin solution, as well as careful observation and subculturing of selected emerging colonies. Young, mature and healthy leaves were also collected, cleaned and surface-sterilized before selected fragments were taken and transferred onto PDA plates. Subsequent processing was as described for stems. Berries were also collected and treated similarly as described for leaves, but only the inner parts of each fruit were plated.

### 2.2. Morphology and Cultural Studies

Isolates were mounted in 60% lactic acid and observed under a light microscope (Olympus BX51, Tokyo, Japan) fitted with a differential interference contrast light and a digital image capture system (Olympus Q-Color 3™ camera, Canada).

Additionally, a slide-culture method [28] was used for selected isolates. Small blocks of oatmeal agar (OA) were inoculated on their sides, and a sterile coverslip was placed on top of each block. These blocks were transferred to an incubator, adjusted to 25 ± 2 °C under a (12 h/12 h) daily light regime (light provided by two white fluorescent daylight bulbs, FLC, 25 W−127 V, placed 35 cm above the plates), for 5–6 days. Subsequently, the agar blocks were removed, and the coverslips and slides, bearing the fungal cultures, were mounted (as described in [28]) for further examination.

Morphological data of relevance for species delimitation in *Clonostachys* and related taxa—shapes of conidiophores, sizes of stipe and penicillus and shapes and sizes of phialides and conidia, among others—were recorded for at least thirty representative structures. Photo images were prepared in Inkscape 1.2.2 (https://inkscape.org/pt/ (accessed on 1 August 2019)).

Colony characters were recorded after 10 days of growth on potato dextrose agar (PDA), OA and 3% malt extract agar (MEA) [29], at 25 ± 2 °C under a 12 h/day light regime. Colony morphology was described based on the standard terminology [30]. Colony color terminology followed that of Rayner [31]. Each isolate was replicated on three separate plates. Colony diameter was measured after 10 days of incubation under the conditions described above.

Eight out of over 1500 isolates obtained during the surveys were assigned as Clonostachys-like. These isolates were either stored on potato carrot agar (PCA) slants at 4 °C for short-term use or, for long-term storage, kept at −80 °C in cryotubes with 10% glycerol, as described in the literature [32]. Selected isolates (Table 1) were deposited in the culture collection of the Universidade Federal de Viçosa (UFV), Viçosa-MG, Brazil-Coleção Octavio de Almeida Drummond (COAD), which is an internationally recognized culture collection registered in the World Federation for Culture Collections.

In order to document the colonization of uredinia by *Clonostachys* spp., pieces of samples of coffee leaves obtained at the end of the in planta antagonism study (described below) and bearing CLR pustules seemingly colonized by Clonostachys were selected and dried in a plant press. Selected pieces of leaves containing mycoparsitized uredinia were further dried via mounting on stubs with double-sided adhesive tape and leaving overnight in a desiccator. These specimens were gold-coated using a Balzer’s FDU 010 sputter coater. A Carl-Zeiss Model LEO VP 1430 scanning electron microscope (SEM) was used, operating at 10 Kv and with a working distance ranging from 10 to 30 mm, to analyze the specimens and generate representative electromicrographs of the colonization events.

### 2.3. DNA Extraction, Polymerase Chain Reactions (PCRs) and Sequencing

Isolates recognized morphologically as belonging to *Clonostachys* were further analyzed molecularly. Four gene regions were sequenced for those isolates, following the methodology described above: namely, TEF1-α (translation elongation factor 1 alpha) [33,34], ACL1 (ATP citrate lyase), RPB1 (the largest subunit of RNA polymerase II) and TUB (β-tubulin) of the rDNA gene.

The isolates were grown on PDA and then seeded in small plates containing 5 mL of potato dextrose (PD) broth each and incubated for 5 days at 25 °C under a 12/12 h daily light/dark regime. After that period, the mycelial material was removed from the plates and air-dried on sterile filter paper at room temperature for 24 h. Dried mycelial material from each isolate was then transferred to sterile tubes for DNA extraction. Extraction was performed with the Wizard Genomic DNA Purification Kit (Promega, Madison, EUA). Manufacturer’s guidelines were strictly followed. The primer pair of EF1-728F/EF2 was used for amplification and sequencing of the TEF1-α region, acl1-230up/acl1-1220low was used for ACL1 [35], Fa/R8 was used for RPB1 [36] and T1/T2 was used for TUB [37]. The PCR reactions were performed in a total volume of 12 µL as follows: 1 µL of genomic DNA of a concentration of 30 ng/µL, 1 µL (0.5 µL^−1^) of BSA, 0.5 µL (0.01 µg/µL) of DMSO, 0.5 µL of each primer, 2.5 µL of Water MilliQ and 6 µL of Dream Taq. The cycling conditions used during PCR for β-tubulin were set according to the published description [29]. However, the protocol for ALC1 was modified; initial denaturation was at 94.0 °C for 5 min, followed by 38 cycles at 94.0 °C for 30 s, 56.0 °C (annealing temperature) for 30 s and 72.0 °C for 30 s, with a final extension of 7 min at 72.0 °C. For RPB1, initial denaturation was at 94.0 °C for 5 min, followed by 38 cycles at 94.0 °C for 30 s, 56.0–50.0 °C (annealing temperature) for 30 s and 72.0 °C for 30 s, with a final extension of 7 min at 72.0 °C. For TEF1-α, initial denaturation was at 94.0 °C for 5 min, followed by 38 cycles at 94.0 °C for 30 s, 54.0 °C (annealing temperature) for 30 s and 72.0 °C for 30 s, with a final extension of 7 min at 72.0 °C. The amplicons were analyzed on GelRed™ (Thermo Fisher Scientific) and visualized under UV light to verify their size and purity. Then, they were purified with ExoSAP-IT™ (Thermo Fisher Scientific, Waltham, MA, USA), and the PCR products were sequenced by Macrogen Inc., Seoul, South Korea (http://www.macrogen.com, (accessed on 1 August 2019)).

### 2.4. Sequences—Phylogenetic Analyses

In order to assemble and edit the nucleotide sequences, DNA Dragon 1.7.3-DNA Sequence Contig Assembler Software developed by SequentiX-Digital DNA Processing (https://www.sequentix.de/ (accessed on 19 March 2020)) was utilized [38]. BLASTn searches of Genbank were performed through the MegaBlast program to verify the taxonomic and locus identities of the consensus sequences (https://blast.ncbi.nlm.nih.gov/Blast.cgi (accessed on 19 March 2020)). Sequences of reference for species with high identity percentages were selected for alignment according to the same gene region used to amplify the gDNA of the respective isolate. The Muscle algorithm, from the software Aliview, version 1.26 [39], was used to align sequences. Data sets from individual genes and the multilocus combination (ACL1, RPB1, TEF1-α and TUB) data set were investigated using the programs RAxML-HPC on XSEDE 8.2.12 for the maximum likelihood analysis and MrBayes on XSEDE 3.2.7a for the Bayesian inference analysis, both via the CIPRES web portal [40]. Maximum likelihood (ML) analysis was performed with 1000 bootstrap samples.

Bayesian inference (BI) analyses were launched after definition of the best nucleotide substitution model for each gene. JModeltest 2.1.10 software [41] was applied, and different models were selected according to the corrected Akaike information criteria (AICc). The likelihood settings from best-fit evolution model TrN+G were used for ACL, those from TIM1ef+I were used for RPB1, those from TIM3ef+I were used for TEF1-α and those from HKY+G were used for TUB. Phylogenetic analyses were conducted with alignments of 44 parsimony-informative positions at 44/821 bp for ACL, 46/1027 bp for RPB1, 46/563 bp for TEF1-α and 42/562 bp for TUB, following the standard configuration of two runs and four chains for each run. Two independent analyses were run for 20 × 106 generations, and chains were sampled every 1000 generations for each data set. A 0.25 fraction of the initial trees were discarded as burn-in before construction of consensus tree. The average standard deviation of split frequencies (ASDSF) was evaluated for the assessment of the convergence between independent runs and automatically stopped when a determined ASDSF value was reached. A BI concatenated tree with the four gene regions was also constructed with MrBayes under the previous four best-fit models and following the same previous standard configuration via the CIPRES web portal [40]. Clonostachys pseudochroleuca, CBS 192.94T, was included as the outgroup. The tree topologies that resulted from both the BI and ML methods were visualized and compared, and the phylogram thereof was edited in FigTree v1.4.4 (http://tree.bio.ed.ac.uk/software/figtree (accessed on 19 March 2020)) and Inkscape.

Sequences of this study, partially deposited in the NCBI database (GenBank) (http://www.ncbi.nlm.nih.gov/genbank (accessed on 19 March 2020)), and those from different genes (ACL1, RPB1, TEF1-α and TUB) retrieved from GenBank, are listed in Table 1.

### 2.5. In Planta and In Vitro Antagonism of Isolates of Clonostachys against Hemileia vastatrix

#### 2.5.1. Assay Settings

All experiments were conducted twice inside a greenhouse, with partial temperature control and temperatures that ranged between 25 and 32 °C, during the assays.

Healthy four-month-old shade-house-grown coffee plants, *C. arabica* cv. Catuaí-vermelho (IAC 144), with 4 to 5 pairs of fully differentiated leaves each were utilized. The substrate used for cultivation of test plants was a mixture of heat-disinfected soil and rice colonized by the antagonist (and prepared as described below).

In planta antagonism of *Clonostachys* isolates against *H. vastatrix* was tested between Dec 2019 and May 2020 (assay I) and between Jun and Nov 2020 (assay II). The assays were each composed of nine treatments, including the control (plants untreated with any antagonist). Each treatment had five repetitions, and each repetition was represented by one young coffee plant grown in a black polyethylene bag containing 50 g of rice, colonized by an isolate of *Clonostachys* spp., mixed with heat-disinfected soil. Both assays I and II included a total of 45 coffee plants each.

#### 2.5.2. Inoculum Preparation and Application

*Hemileia vastatrix*—Fresh, viable urediniospores were obtained as described by Salcedo-Sarmiento [42]. Twelve young and healthy six-month-old coffee plants (cv. Caturra) were hand-spray-inoculated with a spore suspension of race II of *H. vastatrix* (1 × 10^5^ spores/mL), suspended in 0.05% Tween 20, until runoff and placed in a dew chamber (conditions as described by Salcedo-Sarmiento [42]). After 30–45 days, abundant orange sporulation appeared on the undersides of the leaves. Urediniospores were collected and either preserved as described by Salcedo-Sarmiento [42] or used immediately after harvest for the preparation of the spore suspension for application. Only batches of urediniospores with at least 80% viability were used. Viability was evaluated as described by Salcedo-Sarmiento [42].

*Clonostachys* spp.—All isolates were grown on OA plates at 25 °C under a 12 h light regime until the plates were fully colonized. Ten milliliters of sterile distilled water (SDW) were then added to each of the plates, and the surface of the colonies was scraped with a rubber spatula in order to produce a concentrated suspension of mycelium and conidia. The contents of the plates were then aseptically transferred to 125 mL flasks, each containing 20 mL of 2% malt extract broth (MEB). Each flask was seeded with a single *Clonostachys* isolate. The flasks were left on a lab bench at room temperature for three days and manually agitated daily. After this period, 3 mL of the colonized liquid medium was aseptically transferred to a polypropylene bag (12 × 25 cm) containing 50 g of parboiled rice, 0.22 g of CaCO_3_ and 40 mL of distilled water, which had previously been autoclaved at 121 °C for 20 min. For each treatment (an isolate), five polypropylene plastic bags were prepared. The bags were then placed in a growth room at ca. 22 °C under a 12 h light regime (light provided by two white fluorescent daylight bulbs, FLC, 25 W–127 V; and one near-UV lamp, SCT, 28 W–127 V, placed at least 35 cm away from the bags) and incubated for 10 days. Every two days, the bags were rolled, and their contents were hand-squeezed to avoid formation of large rice/mycelial aggregates and to allow for good aeration in order to stimulate sporulation. Bags containing only autoclave-sterilized, noninoculated parboiled rice were used to treat control plants.

In the second round of production of *Clonostachys* inoculum, each isolate was grown on OA plates in an incubator adjusted to 25 °C and with a 12 h light/12 h daily regime for five days. The bags were then aseptically seeded in a laminar flow cabinet with 5 mm-diameter plugs (six per bag) taken from the margins of the actively growing colonies.

Depending on the intensity of the sporulation observed for each *Clonostachys* isolate, six to ten grams of colonized rice were suspended in 0.05% Tween 20 and placed in 125 mL flasks on a shaker at 25 °C at 130 rpm for 20 min in order to release the conidia from the substrate. The contents of the flasks were then filtered through cheesecloth, and the concentrations of conidial suspension were then adjusted with a haemocytometer to 10^6^–10^8^ conidia/mL before use.

Inoculation procedures—In total, 250 g of parboiled rice colonized by each isolate of *Clonostachys* (prepared as previously described), with an estimated 10^7^–10^9^ conidia/g of rice, were thoroughly mixed separately with heat-disinfected soil. This inoculum–substrate mixture was then transferred to five 24 × 17 × 10 cm plastic bags, each containing one healthy young coffee plant. Controls consisted of plants treated as previously described but receiving only a mixture of uncolonized rice.

Spraying of the antagonists on the aerial parts of the coffee plants was conducted one month after soil inoculation with colonized rice (as described above). The conidial suspensions of each isolate were applied separately, until runoff, using a handheld sprayer connected to an air compressor. The foliar sprayings were repeated three times at monthly intervals.

Inoculation of the rust fungus was through spray-inoculation of urediniospores with 2 × 10^5^ spores/mL until runoff. Inoculation with *H. vastatrix* occurred 72 h after the last spray-inoculation with the isolates of *Clonostachys* (3 months after transplantation) and was conducted in the late evening in order to favor spore germination and host infection.

#### 2.5.3. Dual Purpose Assay: In Planta Antagonism of *Clonostachys* against *Hemileia vastatrix* and Evaluation of Endophytic Establishment in *Coffea arabica*

Isolates of *Clonostachys*, originally obtained as endophytes (three) and mycoparasites (five), were used. In order to further confirm their ability to grow as endophytes, coffee plants in the in planta antagonism experiment were also used to evaluate the endophytic ability of all isolates. Two similar (pseudorepeat) assays were then conducted to verify (a) the establishment of the isolates as endophytes in Arabica coffee after inoculation and (b) if an anti-CLR antagonistic “bodyguard-effect” resulted from each isolate application.

Inoculation of young *C. arabica* cv. Catuaí-vermelho with each *Clonostachys* isolate involved a combination of soil application and a conidial suspension spray of the aerial parts (as described previously). Thirty days after transplantation of the plants, the aerial part of each treated group was separately spray-inoculated with a suspension of each *Clonostachys* isolate (10^6^–10^8^ conidia/mL).

Assay I: endophytic colonization. Thirty days after transplantation of the coffee plants, one plant was arbitrarily selected from each isolate-treated group and one from the untreated control group for the endophyte-isolation protocol. This was repeated at 30-day intervals, prior to the round of spraying of antagonists on the aerial part of each plant group. The final round of endophyte isolation was conducted after the final assessment of CLR severity (that is, 5 months after the coffee plants had been transplanted to larger bags). The plants that showed the lowest degrees of CLR severity in each treatment were selected.

The endophyte-isolation protocol was as follows: each individual plant was thoroughly washed under a tap to remove all debris and treated according to a modified version of the protocol of Rodríguez [22]. Each coffee-plant tissue (roots, stems and leaves) was detached using flame-sterilized tools. Root and stem fragments were cut into 2 cm-long pieces, and 5 mm-diameter leaf disks were taken from selected healthy leaves (at the bottom, middle and top of the young plant) with a flame-sterilized cork-borer. Stem pieces had their bark removed before disinfection. Disinfection of each plant part was performed through a sequence of immersions: namely 70% ethanol (1 min), 2% sodium hypochlorite (3 min) and 70% ethanol (1 min). This was followed by rinsing three times with SDW and placing the pieces on sterilized filter paper under aseptic conditions for the removal of excess water. Ten pieces of each tissue of each plant were then plated separately on OA plates supplemented with antibiotics (chloramphenicol and penicillin-G) in order to exclude possible endophytic or contaminant bacterial colonies. Three plates were used for each plant tissue, each then sealed with plastic film and placed in a growth chamber for two to three weeks at 25 °C under a daily 12 h light regime (light conditions as above). After autoclaving at 121 °C for 15 min and cooling (40–50 °C), 1 mL of each antibiotic was aseptically added to the medium (1/100 mL). This volume was taken from stock solutions of individual antibiotics prepared at 1 g/100 mL.

The plates were observed every two days in order to monitor the presence or absence of typical *Clonostachys* colonies. Whenever colonies similar to *Clonostachys* appeared, the plates were further checked under a dissecting microscope (Olympus SZX7). If needed, the plates were opened in a laminar flow and samples of a colony were mounted on microscope slides in a drop of lactofuchsin and examined under a light microscope (Olympus BX-51). The final results were ranked either as positive (+) for recovery of *Clonostachys* growing endophytically in tissues or negative (−) for not a single sample of that tissue producing a *Clonostachys* colony.

Assay II—endophytic colonization. This assay was repeated with all eight isolates of *Clonostachys*. Procedures were mostly as described for assay I. The main difference between assays I and II was the limitation of attempts to confirm the endophytic colonization of *Clonostachys* isolates in a single round in assay II. This was performed at the end of the assessment of CLR severity, five months after the first inoculation of the antagonists and at the end of the greenhouse experiment.

Assay I—in planta antagonism to *Hemileia vastatrix* (as reflected by the effect on CLR severity). Antagonism of different isolates to *H. vastatrix* was tested on coffee plants, in parallel with the test described above. These assays followed the steps described above, but the procedures for assays I and II differed. In order to assess the effects of different isolates, all plants (including the controls) were inoculated with *H. vastatrix* and grown as described above. CLR inoculation was conducted only once, at the third month after the transplantation and 72 h after the third foliar application of the conidial suspensions of *Clonostachys* isolates. This assay involved all 16 remaining plants, representing the eight *Clonostachys* treatments, and two plants for the control treatment (two plants for each treatment).

Three weeks after inoculation with *H. vastatrix*, eight leaves per plant, which showed typical pale yellow spots (an initial symptom of CLR) and were identified with plastic tags, were arbitrarily selected. A total of sixteen leaves from each *Clonostachys*-isolate-treated plant and sixteen control leaves not treated with *Clonostachys* isolates were labeled. Each individual plant represented an experimental unit. The median of the CLR severities evaluated from the eight leaves per plant was recorded as the CLR severity of the plant. One month after urediniospore application, the first CLR severity assessment of individual leaves was performed. CLR severity on individual leaves was assessed three times at two-week intervals. Each leaf was given a note following the standard protocol described by Belan [43], which comprises seven levels of CLR severity, ranging from 0.0 to 50.9%, where 0.0% indicates a complete absence of sporulation and 50.9% the maximum disease severity level.

Assay II: in planta antagonism to *Hemileia vastatrix* (as reflected by effect on CLR severity). As previously mentioned, no attempt at recovering *Clonostachys* from roots, stems or leaves was attempted in assay II until the end. Therefore, all five of each *Clonostachys*-isolate-treated coffee plant and five control plants were included in the CLR severity evaluation. Evaluation of disease severity followed the same procedure as described above. A total of 40 leaves, in the control and each *Clonostachys*-treated plant were marked and evaluated. Evaluation of disease severity for individually marked leaves was conducted three times, as described for assay I. Nevertheless, only the data collected from the third disease severity assessment were statistically analyzed in this study due to the single cycle of the disease under our experimental conditions.

#### 2.5.4. Inhibition of *Hemileia vastatrix* Urediniospore Germination and Mycoparasitism

Isolates of *Clonostachys* spp. that showed the highest levels of reduction in CLR severity in both experiments, as compared to both the control and other *Clonostachys* isolates (COAD 2982 and COAD 2981 (*C. rhizophaga*) and COAD 2984 (*C. rosea*)), were included in one additional test. This test consisted of calculating the in vitro inhibition of germination of urediniospores. Suspensions of spores and filtrates of each isolate were tested.

*Clonostachys* isolates were also grown separately in 125 mL flasks containing 75 mL of oatmeal broth and seeded with five plugs of the corresponding isolate taken from five-day-old colonies on OA plates. The flasks were placed in a shaker at 29 ± 1 °C and 196 rpm for one week. Subsequently, the supernatants were filtered through sterile filter paper in a Büchner funnel. Each filtrate was used in the test, as described below. A suspension of *H. vastatrix* urediniospores was prepared from a stock, collected and stored as previously described. Urediniospores were suspended in a 0.05% Tween 20 solution, and the concentration of urediniospores was calibrated to 1 × 10^5^ spores/mL using a haemocytometer. A suspension of each of three *Clonostachys* isolates was prepared from colonized rice suspended in a 0.05% Tween 20 solution, and the conidial concentration was calibrated to 10^6^ conidia/mL with a haemocytometer.

Two microscope slides were cleaned with 70% ethanol and placed inside polypropylene boxes (11 × 11 × 3.5 cm) that had also been fumigated with 70% ethanol. The boxes were lined with a layer of sterilized paper towel saturated with sterile distilled water (SDW). One 15 μL drop of the urediniospore suspension was transferred, with a micropipette, to the center of each slide; a second 15 μL drop of filtrate of a specific isolate was placed over one of the drops of urediniospore suspension on one of the slides inside the box but not on the other; and the two drops were gently mixed with the tip of the micropipette and the box was covered. Any slide with only one drop of urediniospore suspension served as a control. This study involved four replicates for each *Clonostachys* isolate. All 24 boxes were kept in the dark for six hours at 22 °C, after which urediniospore germination was interrupted via adding a 15 μL drop of lactofuschin to each drop and then observed under a light microscope (Olympus BX-51). This protocol was used to test conidial suspensions of each of the selected *Clonostachys* isolates. The number of germinated vs. nongerminated urediniospores was estimated via observing the first 100 urediniospores on each slide. Urediniospores were considered to have germinated when the germ tubes had a length equal to or longer than the spore diameter. Germination inhibition (% GI) was calculated via following this equation:

% GI = (1 − X/C) × 100

where C = germinated urediniospores on a control slide and X = germinated urediniospores exposed to the antagonist [44].

### 2.6. Statistical Analysis

The mean values of the percent of severity of CLR were calculated. Data were submitted to assumptions of normality and homogeneity of variance using the Shapiro–Wilk and Levene tests before the proceeding of the independent one-way analysis of variance (ANOVA). The distribution of the estimated CLR severities was represented in the form of boxplots. The mean values of the treatments were compared to the mean value of the control with Dunnett’s post hoc test (*p* = 0.05), using JASP software, version 0.16.0 (Statistics Program from University of Amsterdam, https://jasp-stats.org/, 15 January 2021).

## 3. Results

### 3.1. Phylogeny

**Figure 1:**
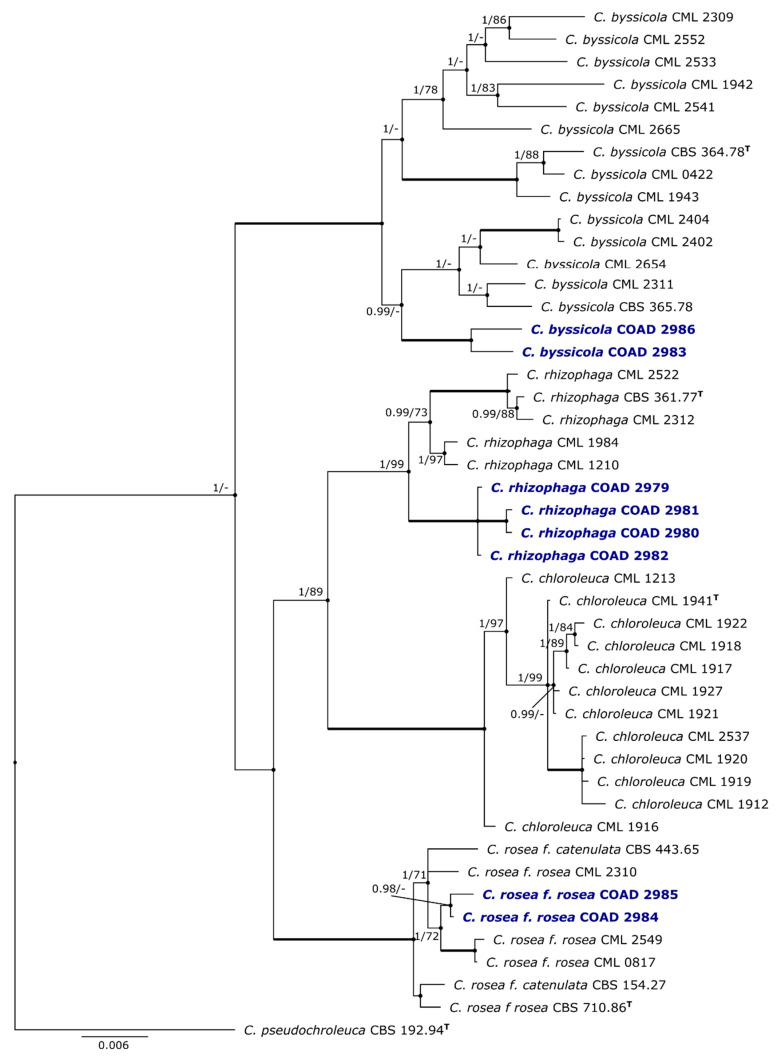
Bayesian inference (BI) based on analysis of a combined data set of *ACL1, RPB1, TEF1-α* and *TUB* sequence data; the concatenated tree is rooted with *Clonostachys pseudochroleuca* (CBS 192.94**^T^**). Branches in bold represent a Bayesian posterior probability of 1.00 and100% bootstrap support for ML, with indicated values at the nodes; the sequences generated in this study are in blue; and branch lengths are proportional to distance. T: ex-type strain; the bar indicates the number of substitutions per site.

BLASTn searches applied to the sequences from the different amplified regions revealed that all isolates belonged to the genus *Clonostachys* (with a threshold of identity ≥ 96%). Amongst these eight isolates, three were isolated as endophytes from stems of *C. arabica*, four were isolated from colonies growing on rust uredinia on *C. canephora* and one was from a colony growing on uredinia on *C. arabica* (Table 1). The BI and ML concatenated trees obtained via combining the available sequences from all four loci (*ACL1, RPB1, TEF1-α* and *TUB*) of species of the genus *Clonostachys* had equivalent topologies. The BI tree is shown in Figure 1. It revealed that the collection of *Clonostachys* isolates from coffee and *Hemileia* uredinia belonged to *Clonostachys byssicola*, *C. rhizophaga* and *C. rosea*. These are well-known taxa that have already been described and discussed in detail in the literature [29,45,46].

Four of the isolates obtained as purported mycoparasites of *Hemileia* uredinia formed a clade with *C. rhizophaga*. Two endophytic isolates grouped within the *C. rosea* clade. The other two isolates—one an endophyte and the other a mycoparasite—formed a clade with *C. byssicola*. The topologies of the single-gene trees (trees not shown) were not all equivalent to that of the concatenated tree. For *ACL1* and *RPB1*, all species strains were monophyletically grouped in both ML and BI analyses. The four isolates in the monophyletic group of *C. rhizophaga* were always divided into two groups; however, in the concatenated tree, they were well-grouped and formed a sister group with the strains already identified as *C. rhizophaga*. In the *TEF1* tree, the strains already identified as *C. rhizophaga* appeared in two separate clades. The same was seen in the *TUB* tree for the *C. byssicola* strains, which also appeared in two separate clades in the ML and BI analyses. However, all isolates of the three species formed monophyletic clades in the concatenated tree, with support values ranging from 70 to 100% ML bootstrap support and BI posterior probabilities of ≥ 0.98 (Figure 1). The sequencing of the amplified *TUB* region within the gDNA of the four isolates of *C. rhizophaga* showed paralogous sequences, thereby being absent in Table 1.

### 3.2. Taxonomy

A comparison of the morphologies of the isolates COAD 2983 and COAD 2986; COAD 2979, COAD 2980, COAD 2981 and COAD 2982; and COAD 2984 and COAD 2985, with published descriptions of *Clonostachys byssicola*, *C. rhizophaga* and *C. rosea*, respectively [45,47], further confirmed their placement within the accepted boundaries of each of these species, as shown in Figure 1 (Table 2).

***Clonostachys byssicola***: Schroers, Stud. Mycol. 46: 80 (2001) [45]. Sexual morphology: *Bionectria byssicola* (Berk. and Broome), Schroers and Samuels, Z. Mykol. 63(2): 152 (1997).

Material examined: Ethiopia, Southern Nations, Nationalities and Peoples Region, Kaffa Zone, Bonga District, Komba Wild Forest Reserve, cloud forest, 2000 m; isolated as a mycoparasite on uredinia of *Hemileia* cf. *coffeicola* (COAD 2983) and as an endophyte in stems of wild *Coffea arabica* (COAD 2986), 25 November 2015, Evans H.C. and Bekele K.B.

For a detailed description and illustrations, see Schroers [45].

**Figure 2:**
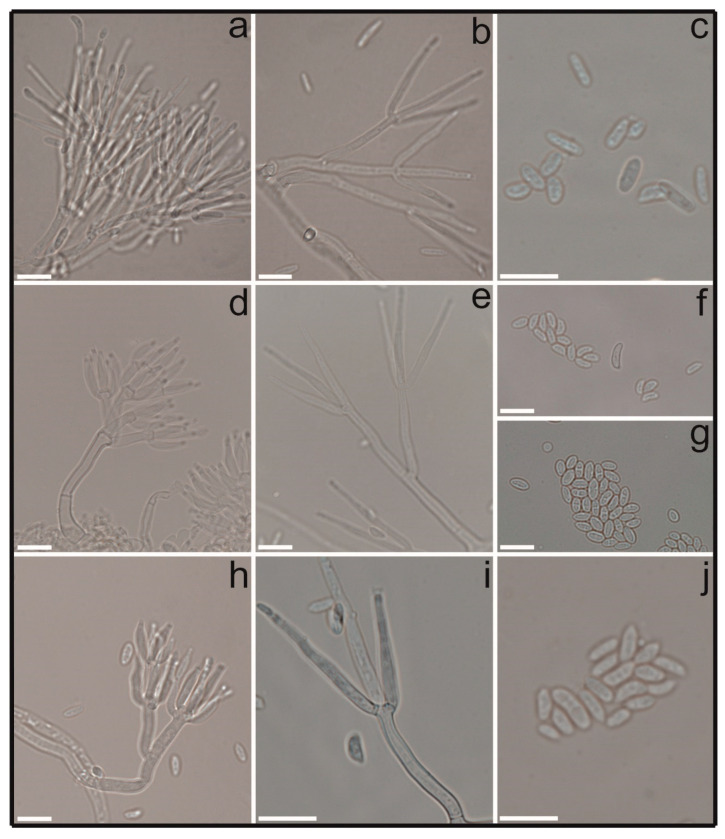
*Clonostachys* spp. obtained during the survey for fungal antagonists of *Hemileia vastatrix*; photomicrographs taken from slide cultures on OA (5–6 days of growth at 25 °C in light/dark-12/12 hs daily light/dark regime). (**a**–**c**) *C. byssicola* COAD 2983: (**a**) secondary conidiophore, (**b**) primary conidiophore and (**c**) conidia; (**d**–**g**) *C. rhizophaga* COAD 2982 and COAD 2980: (**d**) secondary conidiophore, (**e**) primary conidiophore and (**f**,**g**) conidia; (**h**–**j**) *C. rosea f. rosea* COAD 2984: (**h**) secondary conidiophore, (**i**) primary conidiophore and (**j**) conidia; (**a**–**c)** COAD 2983; (**d**−**f)** COAD 2982; (**g**) COAD 2980; and (**h**–**j**) COAD 2984. Scale bar = 10 µm.

Dimorphic conidiophores: verticillate primary conidiophores; divergent phialides (2–4 whorls), 19–54 × 2–3 µm; stipes, 10–63 µm long; penicillus, 19–55.5 µm long; adpressed secondary conidiophores, bi- to triverticillate; adpressed phialides (4–7 whorls), 10–32.5 × 1–2.5 µm. Aseptate conidia: cylindrical and/or slightly curved, 2.5–10 (–14) × 1.5–4 µm (Figure 2).

**Figure 3:**
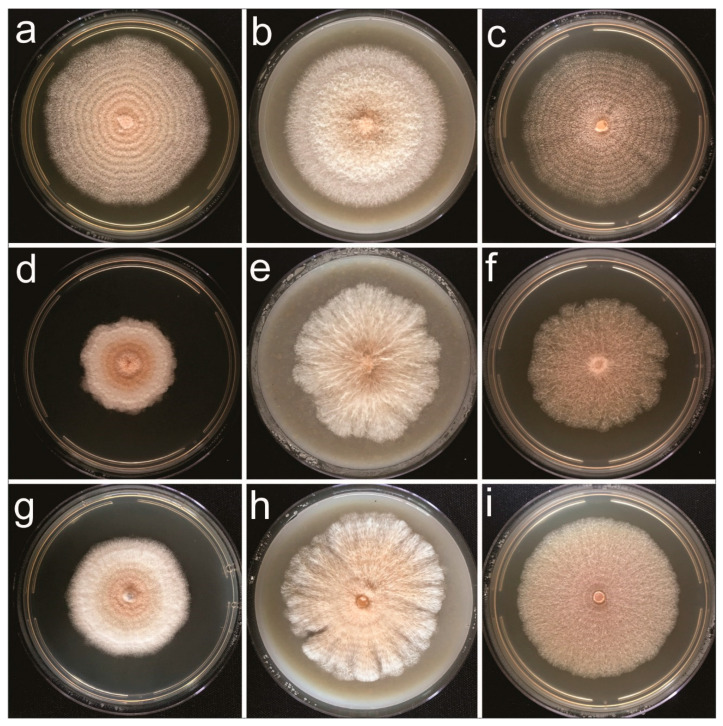
Ten-day-old colonies of *Clonostachys* spp. grown in a 25 °C 12/12 h daily light/dark regime. Left to right, PDA−OA−MEA media: (**a**–**c**) *C. byssicola* COAD 2983 (**d**–**f**) *C. rhizophaga* COAD 2979 and (**g**–**i**) *C. rosea f. rosea* COAD 2985.

In culture (Figure 3): low, convex colonies; entire edge, felty or granular aerial mycelium (due to sporulation); rosy buff; white or pale yellow conidial mass; reverse rosy buff; abundant sporulation. Average colony diameters after 10 days: 67.5 mm on OA, 55 mm on PDA and 64.5 mm on MEA.

*Notes*—When compared with COAD 2983, some morphological details given in [45] for *C. byssicola* were different. Primary conidiophore phialides were shorter (12.4–48 × 1.4–2.8 µm), stipes and the penicillus were longer (10–100 µm and 20–100 µm, respectively); secondary conidiophores had more verticils (2–5) and phialides were in smaller whorls (3–5) and were shorter (7.6–27.8 µm). Such morphological discrepancies between COAD 2983 and the data given in [45] were not regarded as significant for taxonomic separation.

***Clonostachys rhizophaga***: Schroers, Stud. Mycol. 46: 85 (2001) [45].

Material examined: Cameroon, Eastern Province, Somalomo, 700 m; isolated as mycoparasites of uredinia of *Hemileia vastatrix/coffeicola* on leaves of *Coffea canephora*, 22 November 2015, Evans H.C. (cultures: COAD 2979, 2980, 2981 and 2982).

For a complete description and illustrations, see Tehon and Jacobs [48].

Dimorphic conidiophores: verticillate primary conidiophores; divergent phialides (2–5 whorls), (9.5–) 11–35 × 1–3 µm; stipes, 20–92 µm long; penicillus, 19–87 µm long; penicillate secondary conidiophores, mono- to quaterverticillate; adpressed phialides (3–6 whorls), (5−) 14.5–17(−28) × 1 − 2.5(−7) µm. Conidia: aseptate, ellipsoidal/cylindrical, minutely curved to curved, with laterally displaced hilum, 3.5–9 (–11) × 2–5.5 (−7.5) µm (Figure 2).

In culture (Figure 3): flat or effuse colonies, with undulate/fimbriate edges; felty to cottony aerial mycelium, dense and finely granular; white to rosy buff, pigmenting the medium with yellow diffusate; whitish conidial mass; reverse whitish to buff; abundant sporulation. Average colony diameters after 10 days: 66.5 mm on OA, 46 mm on PDA and 40 mm on MEA.

*Notes*—When compared with COAD 2979, some morphological details given in [45] for *C. rhizophaga* differed: primary conidiophores’ penicilli were longer (30–100 µm), and phialides in the secondary conidiophores were narrower (2.2–3.2 µm) in Schroers’ description [45]. Here, we interpreted such discrepancies as representing a variation within the species and not meriting taxonomic recognition.

***Clonostachys rosea***: (Link) Schroers, Samuels, Seifert and W. Gams, Mycologia 91(2): 369 (1999) [47] ***f. rosea***.

Material examined: Ethiopia, Southern Nations, Nationalities and Peoples Region, Kaffa Zone, Bonga District, Komba Wild Forest Reserve, cloud forest, 2000 m; isolated as endophyte from stems of *Coffea arabica*, 25 November 2015, Evans H.C. (Cultures COAD 2984 and 2985).

For a complete description and illustrations, see Schroers [45].

Dimorphic conidiophores: verticillate primary conidiophores; divergent phialides (2–5 whorls), 20.5–40.5 × 1.5–3 µm; stipes, 20–122 µm long; penicillus, 25.5–73.5 µm long; adpressed secondary conidiophores, bi- to quaterverticillate; adpressed phialides (5–7 whorls), 10–19 × 1–3 µm. Conidia: aseptate; cylindrical; less curved; distally broadly rounded, with one slightly flattened side; inconspicuous hilum; conidia size, 4–10 × 1.5–3.5 µm (Figure 2).

In culture (Figure 3): radially striated colonies, with lobate edges; felty aerial mycelium in strands, granulose; white periphery to dense rosy buff towards the center, white conidial mass; reverse whitish to rosy buff; abundant sporulation. Average diameters in 10 days: 67 mm on OA, 53 mm on PDA and 64.5 mm on MEA.

*Notes*—A comparison of the morphology of *C. rosea* f. *rosea*, as described by Schroers [45], with that of COAD 2985 revealed some differences. In [45], the primary penicilli were longer (30–120 µm long), as were the stipes (25–200 µm). The discrepancies highlighted here are interpreted as normal variations within the species.

### 3.3. Dual-Purpose Assay: In Planta Antagonism of Clonostachys against Hemileia vastatrix and Evaluation of Endophytic Establishment

#### 3.3.1. Endophytic Establishment of *Clonostachys* in *Coffea arabica*

Endophytic colonization of the coffee plant tissues by *Clonostachys* isolates was confirmed, albeit with inconsistent results (Table 3).

**Figure 4:**
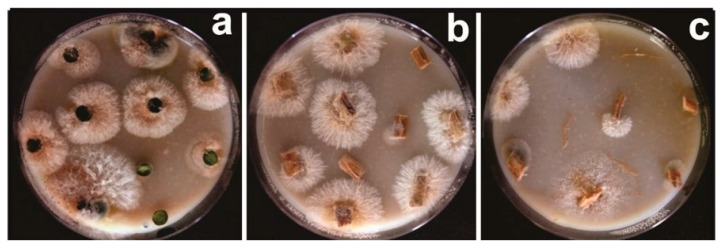
Examples of isolation of *Clonostachys* from coffee tissues inoculated with (**a**) *C. rosea* COAD 2985, from leaf samples; (**b**) *C.rhizophaga* COAD 2979, from stem samples; and (**c**) *C.rhizophaga* COAD 2979, from root samples.

The methodology adopted for this study and the limited duration of the evaluation (for a perennial plant) may be behind such inconsistencies. Consistency in recovery was only observed with plants treated with COAD 2979 in each attempt in assay I and with all treatments, except COAD 2981, two months after the assessment of CLR severity. Conversely, four of the eight treatments (COAD 2980, COAD 2983, COAD 2985 and COAD 2986) yielded a consistent recovery of *Clonostachys* isolates at the end of the experimental period in assay II. The consistency observed with COAD 2979 in assay I during all rounds of recovery was not repeated in assay II. Similarly, the consistency observed with COAD 2982 and COAD 2984 in assay I at the last round of recovery was not repeated in assay II. However, in assay I, all *Clonostachys* isolates treatments were able to colonize at least one tissue of the coffee plants after 30 days of exposure to an inoculum of the antagonist (Figure 4). No *Clonostachys* colonies were obtained from tissues of any of the noninoculated or control plants in both assays.

#### 3.3.2. In Planta Antagonism of *Clonostachys* against *Hemileia vastatrix*

The eight isolates of *Clonostachys* species, i.e., four belonging to *C. rhizophaga*, two to *C. rosea* and two to *C. byssicola*, were screened in planta (Table 1).

**Figure 5:**
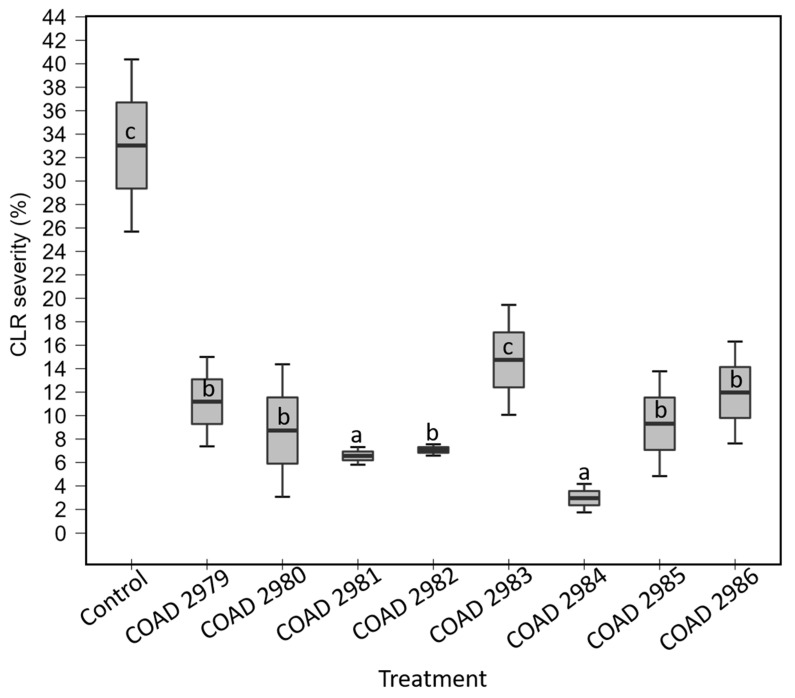
Coffee leaf rust (CLR) severity on *Coffea arabica* plants treated with *Clonostachys* isolates, 60 days after inoculation with *H. vastatrix*, compared with a control not treated with *Clonostachys*. Each mean value is the result of two replicates; means followed by the same letter do not differ significantly (Dunnett, *p* ˂ 0.05). Leaf assessment followed a standard diagram, ranging from 0.0 to 50.9%, the maximum severity level.

In assay I, an independent one-way ANOVA showed a significant effect of treatments on reduction in CLR severity after two months of inoculation of plants with the pathogen; *p* < 0.05. However, post hoc testing using Dunnett’s correction revealed that *C. rhizophaga* (COAD 2981) and *C. rosea* (COAD 2984), at *p* < 0.01, and *C. byssicola* (COAD 2986), *C. rhizophaga* (COAD 2979, COAD 2980, COAD 2982) and *C. rosea* (COAD 2985), at *p* < 0.05, had significantly reduced CLR severity when compared to the control (Figure 5). There was no significant difference between the COAD 2983 treatment (*C. byssicola*) and the control.

**Figure 6:**
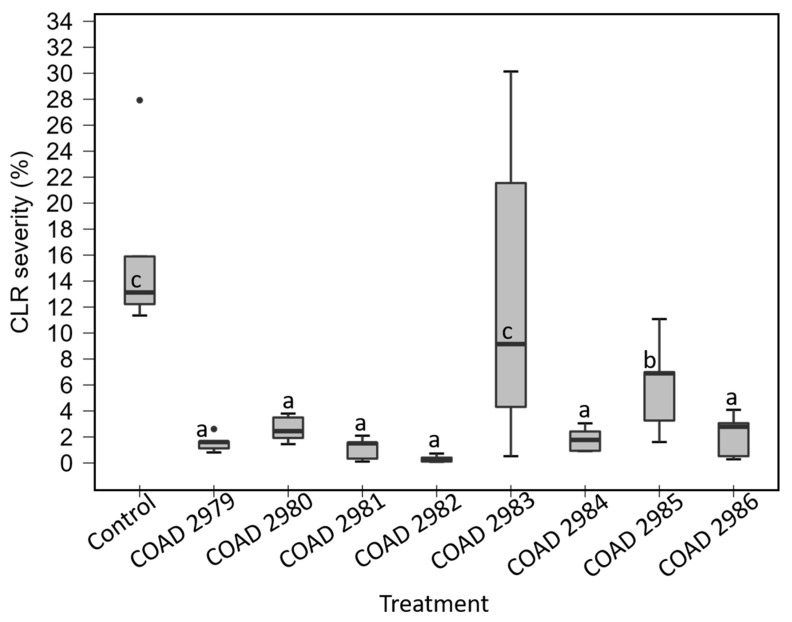
Same as above for experiment II. Each mean value is the result of five replicates; means followed by the same letter do not differ significantly (Dunnett, *p* ˂ 0.05).

In assay II, the independent one-way ANOVA showed a significant effect of treatments on reduction in CLR severity after two months of inoculation of plants with the pathogen; *p* < 0.001. Post hoc testing using Dunnett’s correction revealed that *C. rhizophaga* (COAD 2979, COAD 2980, COAD 2981, COAD 2982), *C. byssicola* (COAD 2986) and *C. rosea* (COAD 2984) were significantly different from the control, at *p* ˂ 0.001, whilst COAD 2985 (*C. rosea*) was significantly different, at *p* ˂ 0.05, from the control in reduction in CLR severity. No significant difference was found between COAD 2983 (*C. byssicola*) and the control (Figure 6).

**Figure 7:**
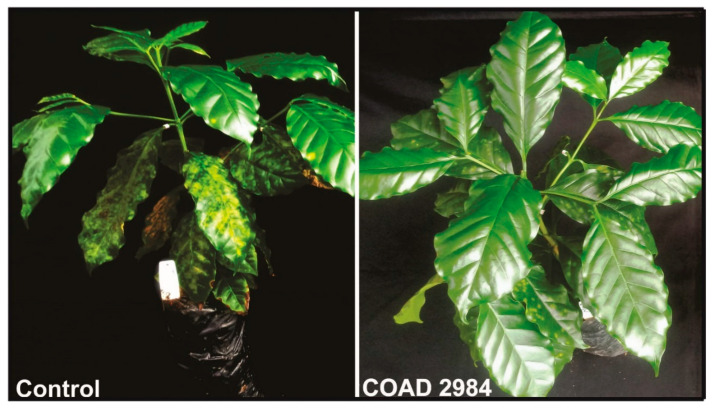
Evidence of reduction in disease severity via *Clonostachys* spp. after 60 days post-inoculation of *Coffea arabica* with *Hemileia vastatrix*: coffee plant grown in soil inoculated with sterile rice, leaves sprayed with sterile distilled water (control); coffee plant grown in soil inoculated with isolate-colonized rice plus sprayed with conidial suspension of *C. rosea* (COAD 2984). Note the smaller number of yellow spots on the adaxial surface in the COAD 2984-treated plant.

Thus, all isolates of *Clonostachys* were able to endophytically colonize *C. arabica* tissues (Table 3), and only COAD 2983 (*C. byssicola*) did not reduce the CLR severity in both experiments (Figure 5 and Figure 6). The evidence of *Clonostachys* spp. reducing the severity of CLR is shown in Figure 7.

### 3.4. Inhibition of Hemileia vastatrix Urediniospore Germination and Mycoparasitism

Isolates of *Clonostachys*—selected for their higher consistency in reducing CLR severity in both assays—were evaluated for their ability to inhibit germination of *H. vastatrix* urediniospores.

**Figure 8:**
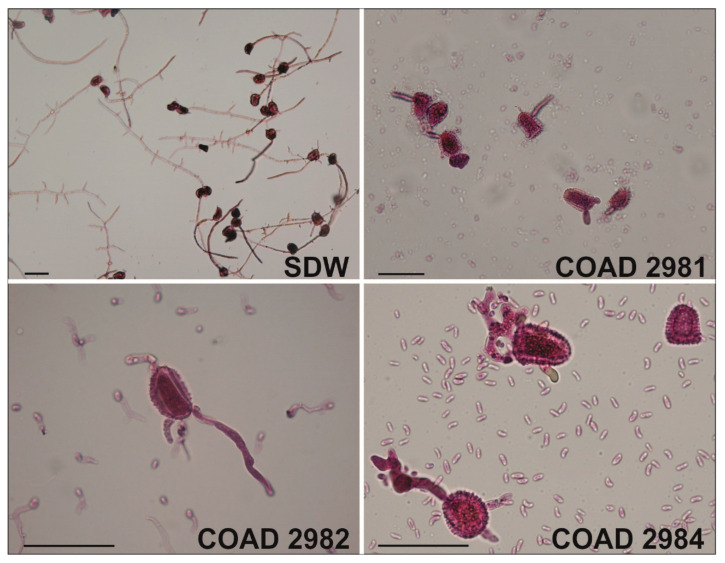
*Clonostachys rhizophaga* and *C. rosea* vs. *Hemileia vastatrix:* effects of suspensions of conidia on germination of urediniospores of *H. vastatrix*. SDW (control): normal germination of urediniospores suspended in sterile distilled water; COAD 2981: inhibition of germination and stubby distortion of urediniospore germ tubes exposed to presence of *C. rhizophaga* (isolate COAD 2981); COAD 2982: ibid. exposure to isolate COAD 2982 of *C. rhizophaga*; COAD 2984: ibid. exposure to isolate COAD 2984 of *C. rosea*. Bars = 50 µm.

Germination of *H. vastatrix* urediniospores after exposure to *Clonostachys* suspensions was drastically reduced and ranged from total inhibition to up to 6%, depending on the *Clonostachys* isolate involved. COAD 2982 (*C. rhizophaga*), COAD 2984 (*C. rosea*) and COAD 2981 (*C. rhizophaga*) inhibited urediniospore germination by 94, 99 and 100%, respectively. Exposure to filtrates of the same isolates resulted in urediniospore germination dropping to 21–40%, depending on the *Clonostachys* isolate involved. COAD 2984 (*C. rosea*), COAD 2982 (*C. rhizophaga*) and COAD 2981 (*C. rhizophaga*) inhibited urediniospore germination to 60, 67 and 79%, respectively. Similarly, to what was shown by Salcedo-Sarmiento [42], distortions of the germ tubes were also observed when *H. vastatrix* urediniospores were exposed to both conidial suspensions and filtrates of *Clonostachys* (Figure 8).

**Figure 9:**
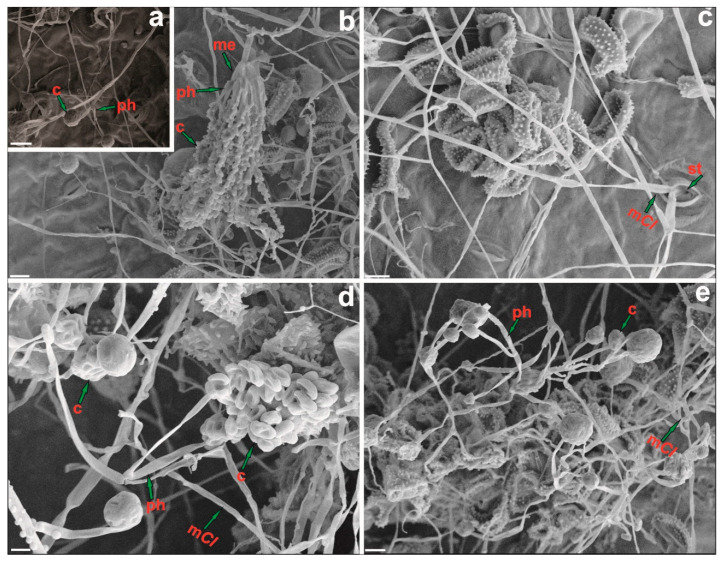
SEM photographs of *Hemileia vastatrix* uredinia inoculated with *Chlonostachys* spp. Asexual structures of *Clonostachys* spp. Note: primary conidiophore (**a**,**e**), secondary conidiophore (**b**,**d**), metulae (me), mycelium of *Clonostachys* (m*Cl*), stomata (st), phialides (ph), conidium. (**c**) Evidence of mycoparasitism with *Clonostachys* spp. on urediniospores of *H. vastatrix*. Invasion of urediniospores by *C. rhizophaga* COAD 2979 (**b**), *C. rhizophaga* COAD 2982 (**d**) and *C. byssicola* COAD 2983 (**e**). Overgrowth of uredinia and evidence of emergence or entrance of *C. rhizophaga* COAD 2980 hyphae through stomata (**c**). Bars = 10 µm.

In addition to the study on microscope slides, an exam for evidence of mycoparasitism was conducted in vivo with selected isolates of *Clonostachys* (COAD 2979, 2980, 2981, 2982: *C. rhizophaga*; and COAD 2983: *C. byssicola*) that had been isolated from uredinia of *Hemileia* rusts. The results from the SEM study also confirmed the mycoparasitic status of *Clonostachys*, showed its ability to colonize pustules of CLR and urediniospores (Figure 9), and also demonstrated the capability of the fungus either to exit the coffee tissue and attack *H. vastatrix* or to spread from colonized pustules back into the plant leaves, as observed in Figure 9c.

## 4. Discussion

*Clonostachys* species have been isolated mainly from soil and litter, but there are also records from bryophytes, wood, bark, black pepper, grapevines, insects, nematodes and even wine [46,49,50]. Some *Clonostachys* species have also been reported as mycoparasites, including *Botrytis cinerea*, an important pathogen that causes gray mold on numerous crops [51,52,53].

Some of those *Clonostachys* spp. have also been investigated as potential biological control agents of several plant pathogens. *Clonostachys byssicola* has been shown to inhibit growth of *Phytophthora palmivora* on cocoa [54,55] and to reduce incidence of banana crown rot [56], whilst *C.* cf. *byssicola* has been evaluated as a biological control agent of *Rosellinia* root rot of cocoa [57]. *Clonostachys rhizophaga* was found to reduce the severity of potato early blight caused by *Alternaria grandis* [58] and has also been reported as a mycoparasite of the chickpea pathogen *Didymella rabiei* [59]. *Clonostachys rosea,* the most broadly studied species for biocontrol purposes, is a well-documented antagonist of *Botrytis cinerea* [51,52,53], *Pythium aphanidermatum* [60], *Phytophthora palmivora* [55] and *Fusarium graminearum* [61].

Research involving *C. rosea* has resulted in development of several biofungicides, including Kamoi, which has been registered for use against gray mold (*B. cinerea*) [62] in Brazil, whilst another strain of *C. rosea* is marketed for control of various soil and seed-borne pathogens [63], and this species has been considered to have enormous commercial potential as a biocontrol agent of plant diseases [64].

Here, we report for the first time the occurrence of members of *Clonostachys* as mycoparasites of *Hemileia* rusts of coffee: namely, *C. rhizophaga* from parasitized uredinia of *H. vastatrix* and *H. coffeicola* on leaves of *C. canephora* in Cameroon and *C. byssicola* from uredinia of *Hemileia* spp. on *C. arabica* from Ethiopia. In addition, the only previous report of a member of *Clonostachys* occurring as an endophyte in coffee tissues was of *Clonostachys* cf. *rosea* isolated from a coffee leaf in Colombia [11]. We isolated both *C. byssicola* and *C. rosea f. rosea* from the inner healthy tissues of coffee stems of *C. arabica* in Ethiopia (Table 1) and, subsequently, demonstrated in greenhouse studies that these isolates can colonize coffee plants and also parasitize *H. vastatrix* (Figure 9).

Isolates of *C. byssicola* (COAD 2983, COAD 2986) and *C. rosea* (COAD 2984 and 2985) proved to be monophyletic, as evidenced by the four genes studied. Nevertheless, some inconsistencies were found for the strains, based on sequences already deposited in GenBank, in the phylogenetic trees of regions such as *TUB* for *C. byssicola* and *TEF1* for *C. rhizophaga*. Trees for these genes (not shown) generated polyphyletic topologies. Similar results (ambiguity for single-gene analysis as compared to consistent monophyly for multilocus analysis of *Clonostachys*) have already been reported in *TUB* for *C. byssicola* [29,47,50] and in *TEF1* for *C. rhizophaga* [29]. Although this occurred for single genes in some strains belonging to *C. byssicola* and *C. rhizophaga*, monophyly was evident in the single-gene trees for *ACL1* and *RPB1*. More importantly, a monophyletic topology was found in the concatenate/multilocus study (Figure 1).

Members of the subclade of the *C. rhizophaga* clade formed of the four mycoparasite isolates of *Hemileia* exhibited contrasting appearances in vitro. All of these isolates were obtained from the same locality in Cameroon. COAD 2981, in contrast to the other three isolates, did not sporulate when cultivated on various media (MEA, OA, PDA) and sporulated poorly when cultivated on autoclaved rice. On OA and PDA, all four isolates released a yellow pigment, which was intense and darker, into the media for COAD 2981 and COAD 2980 (Figure 1). The isolates of COAD 2979, COAD 2980 and COAD 2982 were placed in the same clade and formed abundant secondary conidiophores on OA. These were thought to be rare in *C. rhizophaga*, according to Schroers [45], who was of the opinion that this species represents an “evolutive degeneration” within the genus. In his monograph on the genus [45], Schroers interpreted the abundant production of secondary conidiophores in a particular strain of *C. rhizophaga* (CBS 100004) as representing a feature of a “wild form” of *C. rhizophaga*. In that study, CBS 100004 appeared in the same clade as *C. rhizophaga* CBS 361.77**^T^**, and the latter is included in our study. The production of abundant secondary conidiophores as seen in our isolates, as well as their phylogenetic affinity with CBS 361.77**^T^**, also suggests a “wild state” for our isolates and may reflect their recent isolation from natural forest situations. One observation that could be of relevance for a better understanding of *C. rhizophaga* is the finding of spores that were morphologically similar to the ascospores of the sexual stage of *Clonostachys* in an OA culture of COAD 2980 (Figure 2g), although ascomata were not found and no sexual stage has been reported for this species [45]. Our study suggests that there is significant variability within the taxonomic boundaries of isolates of *C. rhizophaga.*

One of the first steps in any program of biological control is identifying potential biocontrol agents that result from surveys. Our records of *Clonostachys* isolated from *Hemileia* pustules represent novel host associations, and two represent new records of *Clonostachys* growing endophytically in coffee. The subsequent results of our study have proven that these African isolates can successfully establish in coffee plants, mycoparasitize *H. vastatrix* and, therefore, demonstrate potential as biocontrol agents of CLR: the most devastating and economically important coffee disease [5]. Moreover, they can also inhibit in vitro germination of *H. vastatrix* urediniospores and reduce in planta rust severity.

There is published evidence that some mycoparasites can establish long-lasting endophytic associations with plants. For example, Nemec [65] provided evidence of endophytic growth of *Trichoderma harzianum* on sweet orange seedlings, even after eight months of inoculation, and Mejía [66] reported a similar finding for *C. rosea*—a mycoparasite capable of establishing as an endophyte in cacao seedlings. It is difficult, based on our results, to explain the inconsistencies in the isolation of *Clonostachys* from the coffee plant tissues. It can be conjectured that this could have been due to the limited period (five months) covered in this study since the plants were first exposed to *Clonostachys*. Evans [67] also reported similar inconsistencies three months after having inoculated pregerminated cacao beans with endophytic *Trichoderma* isolates.

In summary, all isolates of *Clonostachys* tested here were capable of colonizing coffee as endophytes without inducing disease symptoms, indicating that these isolates are nonpathogenic endophytes that may interact with *C. arabica* in a symbiotic association. This is of particular relevance, since there are records of *Clonostachys* species, including isolates belonging to *C. rhizophaga* and *C. rosea*, being etiological agents of crop diseases: for example, *C. rhizophaga* causing chickpea wilt in Syria [68] and infecting water chestnuts in China [69] and *C. rosea* causing root rot of soybeans in the USA [70] and root and foot rot of faba beans in Iran [71].

*Clonostachys* species have already been reported as potential biocontrol agents against numerous plant pathogens. Some examples are *C. rosea* and *C. byssicola* strains showing evidence of control in vitro of *Moniliophthora roreri*, the causal agent of frosty pod rot of cacao [67]; *C. rosea* reducing sporulation of *M. roreri* on cacao pods [66]; and *Clonostachys* spp. reducing the severity of potato early blight caused by *Alternaria grandis* [58].

It is likely that benign penetration and establishment of endophytes inside *C. arabica* produces changes in the expressions of some genes involved in its defense pathways and the release of potential antimicrobials, such as hydrogen peroxide, peroxidases, ascorbate peroxidase, jasmonic-acid components, pathogenesis-related protein, chitinases, beta glucanases and endochitinases [42,72,73,74,75,76]. *Clonostachys* species are known to produce metabolites and enzymes with a broad spectrum of biological activities, such as antimicrobial/mycoparasitic roles and resistance inducers [77,78]. For example, *Clonostachys rosea* has also been reported to produce cyclopeptides, piperazines, pyranones, sorbicillinoids and clonostach acids [79]. Further investigations along those lines also need to be conducted with COAD 2981 and COAD 2982 (*C. rhizophaga*) and with COAD 2984 (*C. rosea*).

The antagonistic potential shown in the endophytic *Clonostachys* isolates when they were applied to coffee plants before *H. vastatrix* suggests that various mechanisms may be involved: competition for space or nutrients, induced resistance or antibiosis. Guzzo [72] demonstrated that there was a correlation between enzyme-activity increases and systemic protection of coffee leaves treated with *Bacillus thuringiensis* (Thuricide HP). Therefore, further studies would be useful to allow a better understanding of the results reported herein.

Nemec [65] has mentioned that the capacity of a beneficial microorganism to survive in a foreign environment other than its original and to successfully colonize plant roots in the period when protection against pathogens is needed is one of the most important selection criteria for a biological control agent. Based on this assumption, we can postulate that COAD 2979, COAD 2980, COAD 2981 and COAD 2982 (*C. rhizophaga*); COAD 2984 and COAD 2985 (*C. rosea*); and COAD 2986 (*C. byssicola*) form a promising assemblage for further investigation as potential biocontrol agents to be deployed against CLR. However, the combination of the results of the two assays indicates that the list could be further reduced to “potential elites”, prioritizing *C. rhizophaga* COAD 2981 and COAD 2982 and *C. rosea* COAD 2984.

Although the present work is to be regarded as mostly preliminary and exploratory, it has yielded convincing evidence that *Clonostachys* isolates obtained from coffee are capable of growing as endophytes in *C. arabica* and also that some isolates deserve the attention of biocontrol workers who deal with management of CLR. Strictly organized and analyzed experiments, to be performed both under controlled conditions and in the field, are needed and justifiable now. It is hoped that in future studies, some of selected isolates will prove COAD 2981, COAD 2982 and COAD 2984 to be effective as coevolved coffee “bodyguards” with high endophyte competence—as suggested by Muvea [80]—or as high-impact mycoparasites amenable for use as protectants or biofungicides against CLR.

## Figures and Tables

**Table 1:** Details of the *Conostatchys* isolates from coffee and other substrates included in the phylogenetic analysis.

Species	Strain No.	Host/Substrate	Locality/Country	GenBank Accession No.			
				*TEF1-α*	*RPB1*	*ACL1*	*TUB*
*Clonostachys byssicola*	CML 0422	Soil from secondary forest	Benjamin Constant, AM, Brazil	KX184964	KX184899	KX184833	KF871150
	CML1942	Soil from Amazon forest	Benjamin Constant, AM, Brazil	KX184968	KX184903	KX184837	KF871148
	CML1943	Soil from Amazon forest	Benjamin Constant, AM, Brazil	KX184965	KX184900	KX184834	KF871151
	CML2309	*Fragaria ananassa*	Bento Gonçalves, RS, Brazil	KX184966	KX184901	KX184835	KF871149
	CML2311	Parasitizing colony of *Sordaria* sp.	Lavras, MG, Brazil	KX184969	KX184904	KX184838	KF871152
	CML2402	Fruit of *Annona squamosa*	Januária, MG, Brazil	KX184970	KX184905	KX184839	KX185030
	CML2404	Fruit of *Annona x atemoya*	Jaíba, MG, Brazil	KX184971	KX184906	KX184840	KF871153
	CML 2510/CBS 364.78**^T^**	Bark	Venezuela	KX184967	KX184902	KX184836	AF358153
	CML 2511/CBS 365.78	Wood	Venezuela	KX184972	KX184907	KX184841	AF358154
	CML2533	Bryophyte	Itumirim, MG, Brazil	KX184973	KX184908	KX184842	KX185031
	CML2541	Litter	Itumirim, MG, Brazil	KX184974	KX184909	KX184843	KX185032
	CML2552	*Piper nigrum*	Montes Claros, MG, Brazil	KX184975	KX184910	KX184844	KX185033
	CML2654	Litter	Barroso, MG, Brazil	KX184976	KX184911	KX184845	KX185034
	CML2665	Litter	Lavras, MG, Brazil	KX184977	KX184912	KX184846	KX185035
	**COAD 2983**	***Hemileia* sp., *Coffea arabica***	**Bonga, Ethiopia**		**OM038397**	**OM038404**	**OM038391**
	**COAD 2986**	**Endophyte/stems, wild *Coffea arabica***	**Bonga, Ethiopia**		**OM038397**	**OM038401**	**OM038390**
*C. chloroleuca*	CML 2537	Bryophyte	Itumirim, MG, Brazil	KX184989	KX184924	KX184858	KX185038
	CML 1941**^T^**	Native soil from Cerrado	Montividiu, GO, Brazil	KX184988	KX184923	KX184857	KF871172
	CML 1927	Soil under soybean field	Montividiu, GO, Brazil	KX184987	KX184922	KX184856	KF871171
	CML 1922	Native soil from Cerrado	Montividiu, GO, Brazil	KX184986	KX184921	KX184855	KF871170
	CML 1921	Native soil from Cerrado	Montividiu, GO, Brazil	KX184985	KX184920	KX184854	KF871166
	CML 1920	Native soil from Cerrado	Montividiu, GO, Brazil	KX184984	KX184919	KX184853	KX185037
	CML 1919	Native soil from Cerrado	Montividiu, GO, Brazil	KX184983	KX184918	KX184852	KF871167
	CML 1918	Native soil from Cerrado	Montividiu, GO, Brazil	KX184982	KX184917	KX184851	KX185036
	CML 1917	Native soil from Cerrado	Montividiu, GO, Brazil	KX184981	KX184916	KX184850	KF871169
	CML 1916	Soil under cotton field	Montividiu, GO, Brazil	KX184980	KX184915	KX184849	KF871174
	CML 1912	Native soil from Cerrado	Montividiu, GO, Brazil	KX184979	KX184914	KX184848	KF871168
	CML 1213	Native soil from Cerrado	Montividiu, GO, Brazil	KX184978	KX184913	KX184847	KF871173
*C. rhizophaga*	CML 2522	Soil	Lavras, MG, Brazil	KX184994	KX184929	KX184863	KX185039
	CML2514 /CBS 361.77**^T^**	Culture contaminant	Switzerland	KX184993	KX184928	KX184862	AF358158
	CML 2312	Parasitizing colony of *Fusarium oxysporum*	Lavras, MG, Brazil	KX184992	KX184927	KX184861	KF871157
	CML 1984	Native soil from Cerrado	Montividiu, GO, Brazil	KX184991	KX184926	KX184860	KF871155
	CML 1210	Soil under soybean field	Montividiu, GO, Brazil	KX184990	KX184925	KX184859	KF871156
	**COAD 2979**	***H. vastatrix/coffeicola, Coffea canephora***	**Somalomo, Cameroon**		**OM038395**		
	**COAD 2980**	***H. vastatrix/coffeicola, Coffea canephora***	**Somalomo, Cameroon**		**OM038394**	**OM038402**	
	**COAD 2981**	***H. vastatrix/coffeicola, Coffea canephora***	**Somalomo, Cameroon**		**OM038393**		
	**COAD 2982**	***H. vastatrix/coffeicola, Coffea canephora***	**Somalomo, Cameroon**		**OM038396**	**OM038403**	
*C. pseudochroleuca*	CML 2562/CBS 192.94**^T^**	Bark	French Guiana	KX185016	KX184950	KX184885	AF358171
*C. rosea f. catenulata*	CML 2517/CBS 443.65	Soil	USA	KX184996	KX184931	KX184865	AF358166
	CML 2516/CBS 154.27**^T^**	Soil	USA	KX184995	KX184930	KX184864	AF358160
*C. rosea f. rosea*	CML 2549	Litter	Itumirim, MG, Brazil	KX185001	KX184935	KX184870	KX185040
	CML 2518/CBS 710.86**^T^**	Soil, on sclerotia of *Sclerotinia minor*	Netherlands	KX184999	KX184934	KX184868	AF358161
	CML 2310	*Fragaria ananassa*	Caxias do Sul, RS, Brazil	KX184998	KX184933	KX184867	KF871146
	CML 0817	Endophyte, *Lychnophora pinaster*	Ingaí, MG, Brazil	KX184997	KX184932	KX184866	KF871147
	**COAD 2984**	**Endophyte/stems*, Coffea arabica***	**Bonga, Ethiopia**		**OM038392**	**OM038399**	**OM038389**
	**COAD 2985**	**Endophyte/stems*, Coffea arabica***	**Bonga, Ethiopia**			**OM038400**	**OM038388**

In bold: isolates of *Clonostachys* species obtained from *Coffea* spp. during this study. T: ex-type strain.

**Table 2:** Morphologies of *Clonostachys* isolates from coffee and *Hemileia vastatrix* and earlier published descriptions of *Clonostachys* spp. included in the phylogenetic tree.

Character	COAD 2983/2986	*C. byssicola* *	COAD 2979/2980/ 2981/2982	*C. rhizophaga* *	COAD 2984/2985	*C. rosea f. rosea* *
Stipe Length (µm)	10–63	10–100	20–92	10–100	20–122	25–200
Penicillus (µm)	19–55.5	20–100	19–87	30–100	25.5–73.5	30–120
Phialide Shape	divergent	divergent	divergent	divergent	divergent	divergent
Phialides in Whorls	2–4	2–4	2–5	2–5	2–5	2–5
Phialide Size (µm)	19–54 × 2–3	12.4–48 × 1.4–2.8	(9.5)11–35 × 1–3	15.6–48.2 × 2.2–3.2	20.5–40.5 × 1.5–3	25–45 × 1.6–3
Secondary Conidiophore	adpressed	adpressed to divergent	penicillate	penicillate	adpressed	adpressed or divergent
Phialide Size (µm)	10–32.5 × 1–2.5	7.6–27.8 × 1.4–2.8	(5–)14.5–17(–28) × 1–2.5(–7)	5.8–25.2 × 2.2–3.2	10–19 × 1–3	8–18 × 2–3
Phialide Shape	adpressed	±adpressed	adpressed	adpressed or divergent	adpressed	adpressed
Phialides in Whorls	4–7	3–5	3–6	3–5	5–7	–
No. of verticillia	2–3	2–5	1–4	3–4	2–4	2–4
Conidia Size (µm)	2.5–10(–14) × 1.5–4	3.2–10.8 × 1.8–4	3.5–9(11) × 2–5.5(–7.5)	4.8–9 × 2.4–4.2	4–10 × 1.5–3.5	5.6–10 × 2–3.6

* *C. byssicola*, *C. rhizophaga* and *C. rosea* morphological data from Schroers [42].

**Table 3:** Isolation of *Clonostachys* spp. demonstrating endophytic colonization of *Coffea arabica* in different tissues of inoculated vs. (control) noninoculated plants.

Assay I ^a^													Assay II ^b^		
	Month of Isolation (After First Inoculation)														
	1st			2nd			3rd			5th			5th		
Isolate ^c^	Leaf	Stem	Root	Leaf	Stem	Root	Leaf	Stem	Root	Leaf	Stem	Root	Leaf	Stem	Root
Control	− *	−	−	−	−	−	−	−	−	−	−	−	−	−	−
COAD 2979	+ *	+	+	+	+	+	+	+	+	+	+	+	+	+	−
COAD 2980	+	+	−	+	+	+	+	−	+	+	+	+	+	+	+
COAD 2981	+	+	−	+	+	+	−	+	+	+	+	−	+	−	−
COAD 2982	+	+	+	+	+	+	−	−	+	+	+	+	+	+	−
COAD 2983	+	+	+	+	+	−	−	+	+	+	+	+	+	+	+
COAD 2984	+	+	−	+	+	+	+	−	+	+	+	+	+	−	+
COAD 2985	+	+	−	+	+	+	−	−	+	+	+	+	+	+	+
COAD 2986	+	−	+	+	+	+	−	+	+	+	+	+	+	+	+

^a^ Inoculations performed in four rounds in both assays, combining soil application—individual isolates of colonized rice (50 g/plant)—with an estimated 10^7^–10^9^ conidia g^−1^ of rice (first application) and three foliar sprays (conidial suspensions—10^6^–10^8^ conidia ml^−1^ of isolate until runoff) at 30-day-intervals after soil application. Control (noninoculated) plants received 50 g of uncolonized rice per plant and were sprayed with sterile distilled water in parallel with each *Clonostachys*-isolate treatment of the other groups of plants. ^b^ For assay II, a single round of endophyte isolation was performed five months after the first inoculation at the end of the experiment. * + = at least one piece producing a colony of *Clonostachys*; − = no *Clonostachys* colony obtained from any sample after 2–3 weeks. ^c^ COAD numbers represent accession numbers in the culture collection of the Universidade Federal de Viçosa (Coleção Octávio de Almeida Drummond).

## Data Availability

DNA sequence data used in the study are available in the public repository at GenBank.
